# Machine learning-based risk model using ^123^I-metaiodobenzylguanidine to differentially predict modes of cardiac death in heart failure

**DOI:** 10.1007/s12350-020-02173-6

**Published:** 2020-05-14

**Authors:** Kenichi Nakajima, Tomoaki Nakata, Takahiro Doi, Hayato Tada, Koji Maruyama

**Affiliations:** 1 Department of Functional Imaging and Artificial Intelligence, Kanazawa University Graduate School of Medicine, 13-1 Takara-machi, Kanazawa, 920-8640 Japan; 2 Department of Cardiology, Hakodate-Goryoukaku Hospital, Hakodate, Japan; 3 Department of Cardiology, Teine Keijinkai Hospital, Sapporo, Japan; 4 Department of Cardiology, Kanazawa University Hospital, Kanazawa, Japan; 5 Wolfram Research Inc., Tokyo, Japan; 6 Department of Chemistry and Materials Science, Osaka City University, Osaka, Japan

**Keywords:** Risk stratification, artificial intelligence, cardiac mortality, neuroimaging, arrhythmia

## Abstract

**Background:**

Cardiac sympathetic dysfunction is closely associated with cardiac mortality in patients with chronic heart failure (CHF). We analyzed the ability of machine learning incorporating ^123^I-metaiodobenzylguanidine (MIBG) to differentially predict risk of life-threatening arrhythmic events (ArE) and heart failure death (HFD).

**Methods and Results:**

A model was created based on patients with documented 2-year outcomes of CHF (n = 526; age, 66 ± 14 years). Classifiers were trained using 13 variables including age, gender, NYHA functional class, left ventricular ejection fraction and planar ^123^I-MIBG heart-to-mediastinum ratio (HMR). ArE comprised arrhythmic death and appropriate therapy with an implantable cardioverter defibrillator. The probability of ArE and HFD at 2 years was separately calculated based on appropriate classifiers. The probability of HFD significantly increased as HMR decreased when any variables were combined. However, the probability of arrhythmic events was maximal when HMR was intermediate (1.5-2.0 for patients with NYHA class III). Actual rates of ArE were 3% (10/379) and 18% (27/147) in patients at low- (≤ 11%) and high- (> 11%) risk of developing ArE (*P* < .0001), respectively, whereas those of HFD were 2% (6/328) and 49% (98/198) in patients at low-(≤ 15%) and high-(> 15%) risk of HFD (*P* < .0001).

**Conclusion:**

A risk model based on machine learning using clinical variables and ^123^I-MIBG differentially predicted ArE and HFD as causes of cardiac death.

**Electronic supplementary material:**

The online version of this article (10.1007/s12350-020-02173-6) contains supplementary material, which is available to authorized users.

## Introduction

Chronic heart failure (CHF) has become a major public health burden associated with aging of the global population.^1^ Despite significant prognostic improvements due to recent pharmacological therapies and cardiac devices, morbidity and mortality rates remain high; nearly 50% of patients with CHF do not survive beyond 5 years after diagnosis. The conventional prognostic biomarkers of CHF include New York Heart Association (NYHA) functional class, left ventricular ejection fraction (LVEF), blood b-type natriuretic peptide (BNP) or N-terminal proBNP (NT-ProBNP), and influential comorbidities such as diabetes, chronic kidney disease and hypertension.^2^,^3^ Nevertheless, a significant number of patients with CHF cannot benefit from contemporary therapeutic strategies because of exclusion criteria based on current guidelines of heart failure management, cardiac device therapies, or no or minimal responses to therapies. The causes of cardiac death in patients with CHF have been consolidated into progressive pump failure or sudden (arrhythmic) death. Moreover, current guidelines and studies indicate a need to improve risk stratification models for selecting risk-based prophylactic or therapeutic strategies.

Changes in cardiac sympathetic function and innervation assessed by ^123^I-metaiodobenzylguanidine (MIBG) activity comprise prognostic biomarkers for patients with CHF when combined with conventional clinical parameters. Several studies including multicenter investigations have shown that ^123^I-MIBG has powerful ability to predict cardiac mortality risk due to sudden cardiac or pump failure death.^4^-^7^ Some short- or long-term mortality risk models have been created by combining clinical parameters with cardiac ^123^I-MIBG activity.^8^,^9^ We recently validated the significant prognostic value of a risk model to differentiate low- and high-risk populations among a series of patients with CHF.^10^ However, the models could not separate cardiac death due to end-stage heart failure (HFD) and life-threatening arrhythmic events/sudden death (ArE).^11^ The appropriate management of patients with CHF depending on risk of HFD or ArE is of paramount importance, and implantable cardioverter defibrillators (ICD) and cardiac resynchronization therapy (CRT) for patients at high risk of ArE and HFD, respectively, seem reasonable risk-based therapeutic interventions.

This study was designed to establish a means of differentiating the probabilities of cardiac death due to ArE and HFD using a machine learning-based classifier combined with clinical and ^123^I-MIBG parameters, and to evaluate relationship between ^123^I-MIBG activity and events depending on clinical variables. The diagnostic accuracy of the classifier was also determined using training and validation databases that were built based on a cohort of Japanese patients with CHF.

## Methods

This study included 526 patients with CHF who had been consecutively assessed by ^123^I-MIBG imaging at one of four participating hospitals. The patients who had completed follow-up of at least 2 years when lethal cardiac events were not documented within the initial 2 years, were retrospectively selected from the patient medical records. The mean follow-up interval was 30 ± 20 months. Cardiac ^123^I-MIBG studies proceeded between 2005 and 2016, when the patients were clinically stable. No patients had lethal acute myocardial infarction for 2 years. In addition, due to the small number of non-cardiac death events, they were excluded from the statistical analysis so that this study could focus on discriminating ArE and HFD risks. Standard optimal medical care for CHF continued at each hospital after ^123^I-MIBG imaging. We analyzed patient data only when at least 2-year outcomes were confirmed. Among the patients, 77% and 23% had NYHA functional classes I-II and III-IV, respectively, and a mean LVEF of 38% ± 14% determined by two-dimensional echocardiography or gated myocardial perfusion scintigraphy (Table 1).

**Table 1:** Demographics of patients with heart failure

	Mean ± SD (%)	ROC AUC			Model test, *P*	Selection for model
		ArE	HFD	Surviving		
Number of patients (n)	526	37	105	384		
Age (years)	66 ± 14	0.53	0.66	0.62	< .0001	Yes
Male (%)	72%				.049	Yes
NYHA class I/II/III/IV (%)	53/24/18/4%	0.57	0.83	0.79	< .0001	Yes
Estimated glomerular filtration rate (mL·min^−1^/1.73 m^2^)	49 ± 29	0.57	0.63	0.63	< .0001	Yes
Left ventricular ejection fraction (%)	38 ± 14%	0.53	0.60	0.59	.0017	Yes
Hemoglobin (g·dL^−1^)	12 ± 3	0.54	0.63	0.60	.0004	Yes
^123^I-MIBG variables						
Early HMR*	1.90 ± 0.43	0.59	0.73	0.69	< .0001	No
Late HMR*	1.73 ± 0.42	0.57	0.77	0.73	< .0001	Yes
Washout rate	8.6 ± 9.6%	0.56	0.61	0.60	.0029	Yes
BNP and NT-ProBNP categories 0–4 (%)	4/10/13/43/30%	0.59	0.73	0.65	< .0001	Yes
Hemodialysis (%)	14%				.035	Yes
Ischemic etiology (%)	37%				.077	Yes
Hypertension (%)	53%				.015	Yes
Diabetes (%)	40%				< .0001	Yes
Dyslipidemia (%)	35%				.65	No
Medications						No
Beta blocker (%)	85%					No
Angiotensin converting enzyme inhibitor and/or Angiotensin II receptor blocker (%)	70%					No
Diuretics (%)	68%					No
2-year mortality risk (statistical model)^19^	12.2 ± 10.9%	0.60	0.86	0.81	< .0001	No

*ArE*, Arrhythmic event; *BNP*, b-type natriuretic peptide; *HFD*, heart failure death; *HMR*, heart-to-mediastinum ratio; *NT-ProBNP*, N-terminal proBNP; *ROC-AUC*, receiver-operating characteristic analysis-area under the curve

*Standardized to medium-energy general-purpose collimator condition.^14^

### ^123^I-MIBG Study

Patients were injected with 111 MBq of ^123^I-MIBG (FUJIFILM Toyama Chemical Co. Ltd., Tokyo, Japan), then anterior planar scintigrams were acquired 15-30 minutes (early phase) and 3-4 hours (late phase) later at the participating institutions. Standard acquisition protocol was used for ^123^I-MIBG imaging^12^,^13^; 256 × 256-matrix anterior images using a dual-detector SPECT, and energy centered at 159 keV with a 20% window. Cardiac ^123^I-MIBG activity was assessed at each hospital by drawing cardiac and upper mediastinal regions, then calculating heart-to-mediastinum average count ratios (HMR) of ^123^I-MIBG.^13^ Since low-energy high-resolution, low-energy general-purpose, and low-medium-energy collimators were used in four hospitals, we standardized the HMR to medium-energy, general-purpose collimator conditions to adjust for differences among collimators using a phantom-based correction method.^14^,^15^ The ^123^I-MIBG washout rate was calculated using the formula: (early HMR − late HMR)/early HMR.^16^

### Biomarkers

Blood BNP or NT-ProBNP was measured at the participating hospitals. Data were acquired from clinically stable patients around the time of the ^123^I-MIBG study. Because both BNP and NT-ProBNP data were included, these biomarkers were categorically classified for assessment as grades 0, 1, 2, 3 and 4; as follows: BNP < 40, 40-99, 100-199, 200-560 and > 560 pg·mL^−1^, respectively, and NT-ProBNP < 125, 125-399, 400-899, 900-4800 and > 4,800 pg·mL^−1^, respectively.^17^,^18^ Grades 0-3 were based on guidelines, and the highest (grade 4) threshold values were determined by analyzing receiver-operating characteristics (ROC) curves.^10^

### Definitions of Cardiac Events

The primary endpoint of this study was cardiac death due to end-stage heart failure and arrhythmic or sudden cardiac death, which were recorded in the medical records. Sudden cardiac death was defined as witnessed cardiac arrest and death within 1 hour of onset of acute symptoms or unexpected death in patients known to have been well within the previous 24 hours. Appropriate therapies against life-threatening arrhythmic events including ICD discharge and/or anti-arrhythmic pacing, were also included as ArE for patients under therapy with an ICD or a CRT device with a defibrillator (CRT-D).

### Ethics Approval

The Ethics Committees at Kanazawa University and at each participating hospital approved this multicenter study. The need for written informed consent from each patient was waived because of the retrospective nature of this study.

### Statistical 2-Year Mortality Risk Model

We described a statistical four-variable model to assess cardiac death risk in which age, NYHA functional classes I-II or III-IV, ^123^I-MIBG HMR and LVEF were combined.^19^ The calculated mortality rates (%) at 2 years included HFD, sudden cardiac/arrhythmic death and fatal acute myocardial infarction.

### Machine Learning and Modeling of Event Probability

**Figure 1:**
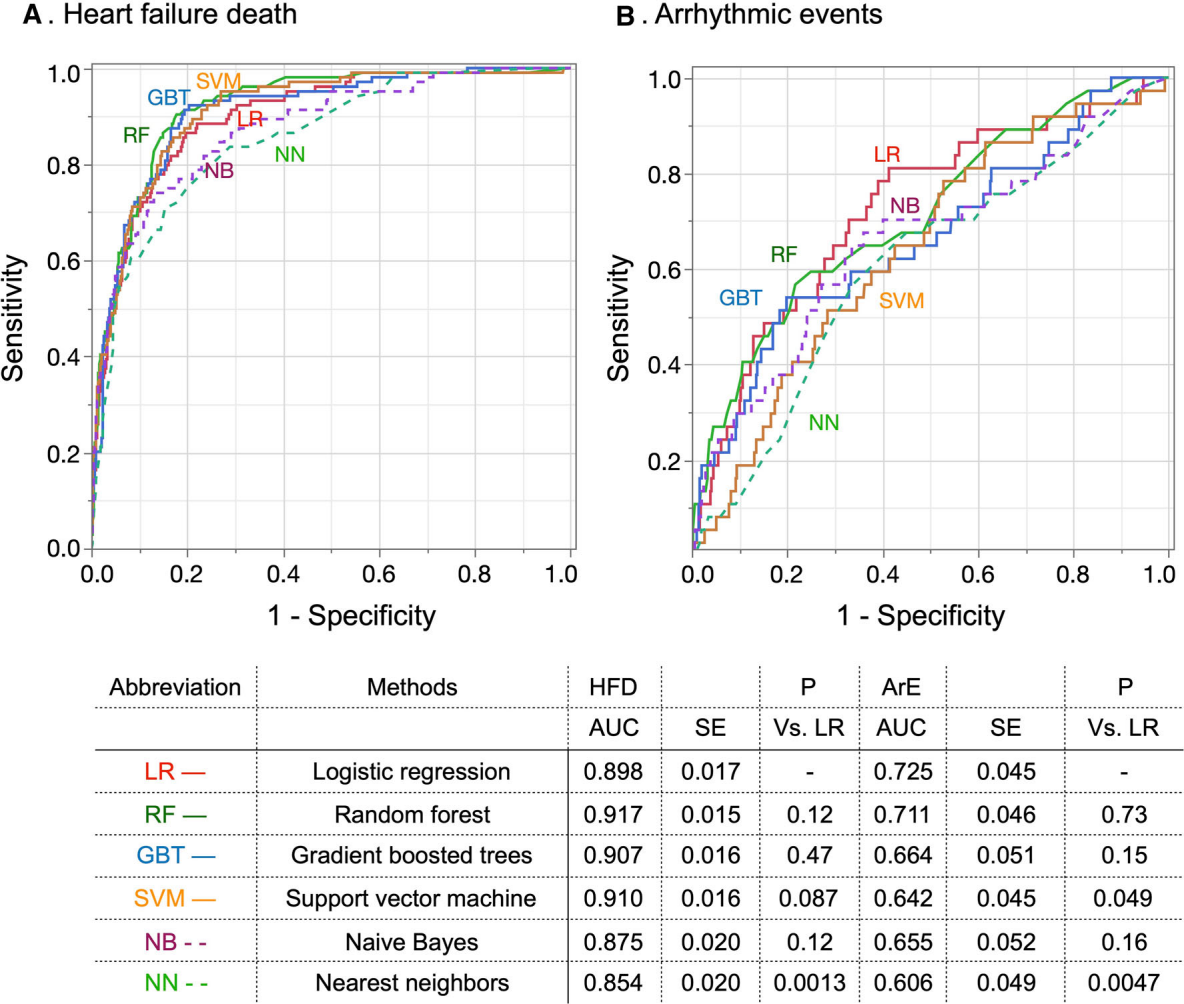
Receiver operating characteristics (ROC) curves of fourfold cross-validation using various machine learning methods

The following 13 variables were selected by ROC analysis: cardiac ^123^I-MIBG indices (late HMR and washout rate), age, NYHA functional class, estimated glomerular filtration rate (eGFR), LVEF, hemoglobin and BNP/NT-ProBNP grade (Table 1). Gender and influencing states such as hemodialysis, ischemic etiology, hypertension, and diabetes mellitus were also included as potential risk factors for events associated with heart failure. The output data comprised three classes of events of HFD, ArE and none (survived). Although various training methods with several optimization strategies were available, we examined areas under ROC curves (AUC) derived from 75% of the patients for training and used the remaining 25% for validation (fourfold cross-validation). As a result, 105 patients with HFD were divided into 78-79 and 26-27 patients for training and validation datasets, and 37 ArE patients into 27-28 and 9-10 patients, respectively. The AUC for logistic regression, support vector machine, gradient boosted trees, random forests, nearest neighbors, and naïve Bayes classifiers are shown in Figure 1. We compared the results of AUC between training and test datasets to avoid the possibility of overfitting. Since the AUC for ArE was best for logistic regression with appropriate regularization, we applied this method to the probability calculation formula for all data. Probability curves were plotted for the ^123^I-MIBG HMR because we calculated the probability that HFD, ArE and no events would occur for each patient using the classifier function. We evaluated the performance of the probability calculation using calibration plots of estimated vs. actual probability. Machine learning was based on Mathematica version 12 (Wolfram Research Inc., Champaign, IL. USA).

### Statistical Analysis

Variables are expressed as means ± standard deviation (SD). Mean values between groups were compared using analyses of variance (ANOVA). Pairs of groups were compared using t tests and contingency analyses, respectively, with Pearson statistics for continuous and categorical variables. The accuracy of the model was determined by calculating the AUC of the dataset. Optimal cutoff values for groups with and without events were determined using the greatest true positive plus true negative rates. Values with *P* < .05 and *P* ≥ .05 were considered significant and non-significant (n. s.), respectively. All data were analyzed using the SAS statistical package JMP version 12 (SAS Institute, Cary, NC, USA).

## Results

**Figure 2:**
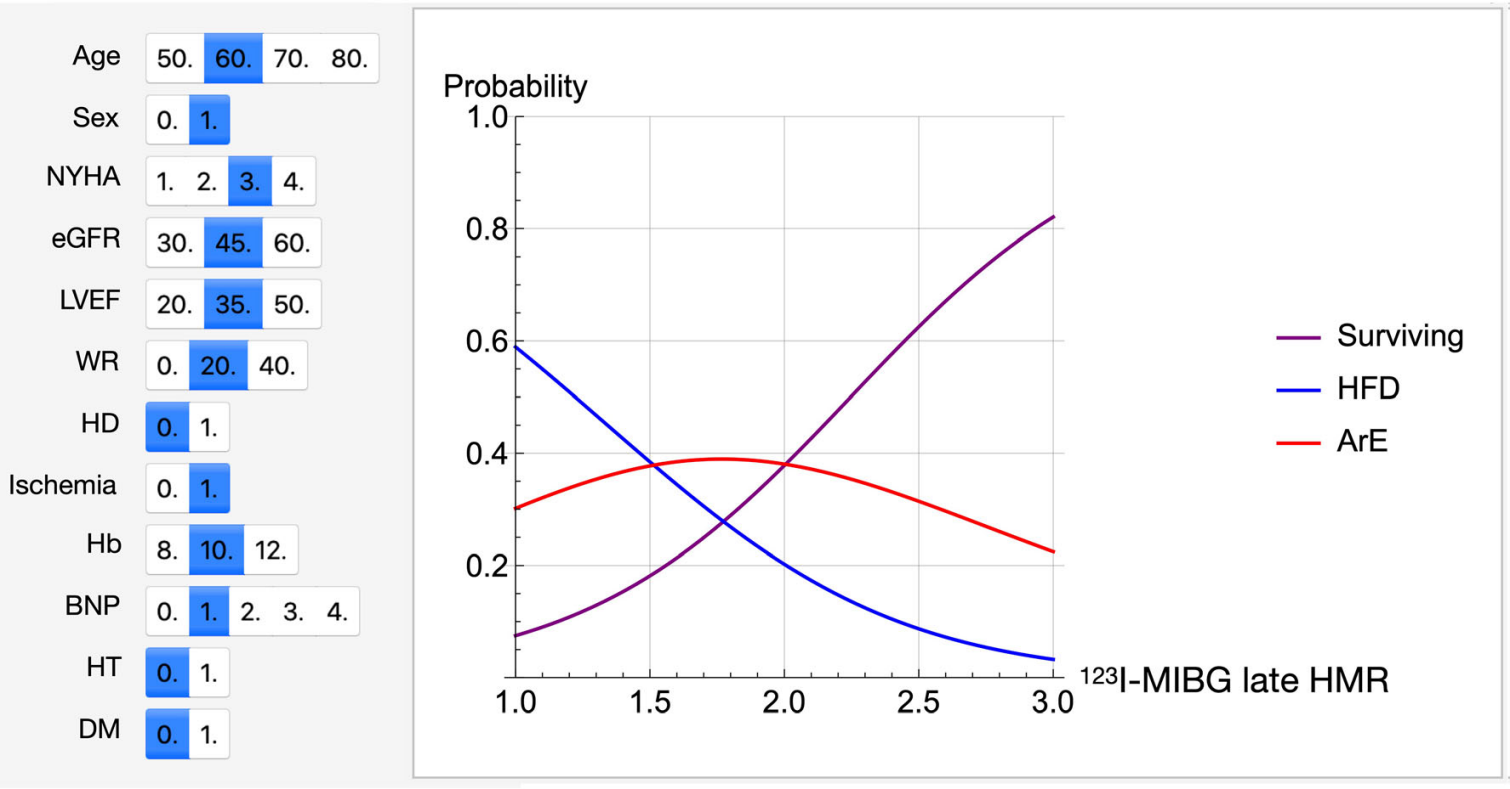
Probability of heart failure death (HFD), arrhythmic events (ArE), survival (no events) against ^123^I-MIBG heart-to-mediastinum ratio (HMR). The probabilities were calculated by a three-category classifier. Selected conditions of the variables are shown in blue

During a 2-year followup, 137 (26%) patients succumbed to cardiac death (HFD, n = 105 [20%]; ArE, n = 32 [6%]). Forty-one patients used an ICD or CRT-D and 12 received appropriate therapy. Arrhythmic events including sudden cardiac death and appropriate ICD/CRT-D therapy occurred in 37 (7%) of 526 patients. Figure 2 shows the probability curves for HFD, ArE and their combination (total cardiac events) relative to HMR on ^123^I-MIBG images as one patient with specific clinical variables that can be selected depending on the characteristics of individual patients, including age, gender, eGFR, NYHA functional class, LVEF, BNP category and ^123^I-MIBG variables.

A comparison of prognostic variables between ArE and HFD (Table 2) showed that patients with HFD were older, had lower HMR, lower hemoglobin, and a higher prevalence of diabetes and hypertension than those with ArE. The rate of actual ArE events was higher in patents with NYHA functional class I-II. Likewise, BNP/NT ProBNP grades were significantly higher in patients with HFD than ArE. The probabilities of ArE and HFD were also estimated better compared with the conventional statistical model for 2-year cardiac mortality.

**Table 2:** Comparison between groups with arrhythmic events and heart failure death

Variables	Fatal arrhythmic events (n = 37)	Heart failure death (n = 104)	*P*
Continuous			
Age (years)	64.7 ± 14.6	72.4 ± 9.8	.0005
LVEF (%)	36 ± 14	34 ± 13	.57
eGFR (mL·min^−1^/1.73 m^2^)	42.9 ± 23.3	39.5 ± 28.7	.51
^123^I-MIBG early HMR	1.86 ± 0.38	1.64 ± 0.34	.0014
^123^I-MIBG late HMR	1.66 ± 0.34	1.45 ± 0.30	.0005
^123^I-MIBG washout rate (%)	10.2 ± 9.8	11.2 ± 8.7	.58
Hemoglobin (g·dL^−1^)	12.6 ± 2.2	11.6 ± 2.2	.014
Probability of all events (%/ 2 y)	39.5 ± 29.4	63.5 ± 26.7	< .0001
Probability of HFD (%/2 y)	23.5 ± 22.8	54.4 ± 26.1	< .0001
Probability of ArE (%/2 y)	16.6 ± 8.6	9.8 ± 7.1	< .0001
ArE/HFD probability ratio	2.8 ± 5.9	0.38 ± 0.62	< .0001
Statistical model of 2-year mortality risk (%)^19^	13.5 ± 2.2	24.8 ± 14.1	< .0001
Categorical			
Sex (male)	78%	80%	.85
NYHA class I/II/III/IV (%)	41/30/24/5%	6/37/44/13%	< .0001
BNP/NT-ProBNP groups 0/1/2/3/4 (%)	3/13/22/46/16%	1/2/3/34/60%	< .0001
Hemodialysis (%)	11%	22%	.13
Ischemic etiology (%)	27%	45%	.053
Hypertension (%)	16%	84%	.014
Diabetes (%)	8%	42%	.0002

*ArE*, Arrhythmic event; *BNP*, b-type natriuretic peptide; *eGFR*, estimated glomerular filtration rate; heart failure death; *HMR*, heart-to-mediastinum ratio; *LVEF*, left ventricular ejection fraction; *NT-ProBNP*, N-terminal proBNP

**Figure 3:**
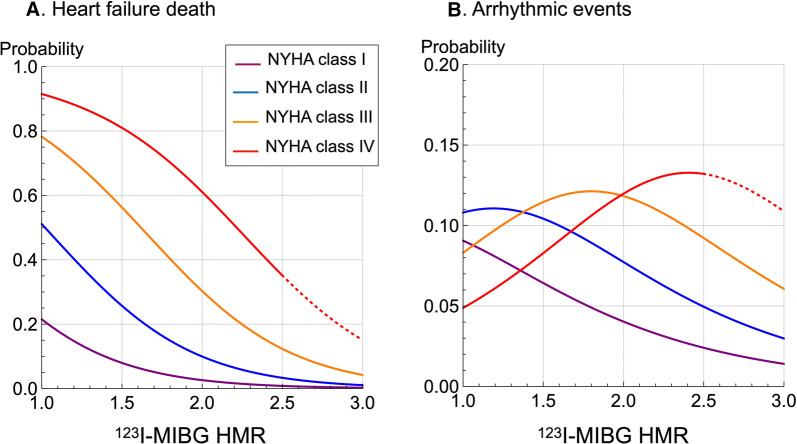
Probability of heart failure death and arrhythmic events vs ^123^I-MIBG heart-to-mediastinum ratio (HMR) in patients with NYHA classes I to IV. Dotted line: Decreased reliability because no patients with NYHA class IV had HMR > 2.5

**Figure 4:**
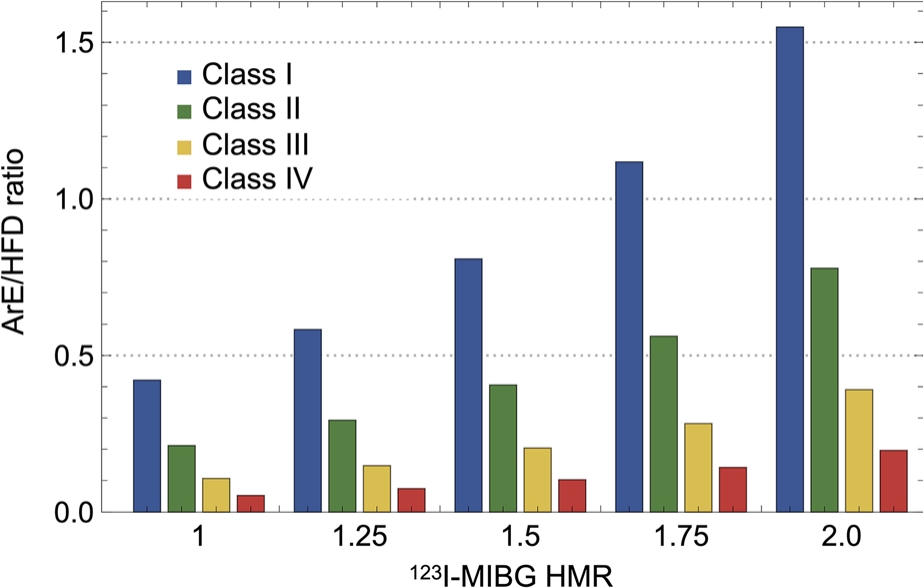
Fraction of arrhythmic event (ArE) probability divided by heart failure death (HFD) probability in patients with NYHA classes I, II, III, and IV vs ^123^I-MIBG heart-to-mediastinum ratio (HMR)

We fixed the remaining variables to each mean value to determine the effects of a single variable on the probability of ArE and HFD. The probability of HFD significantly increased in relation to NYHA functional class but inversely decreased with increasing ^123^I-MIBG HMR (Figure 3A). In contrast, the probability of ArE was the highest at the intermediate range of MIBG HMR, (1.5-2.0 for patients with NYHA class III), showing a bell-shaped probability curve (Figure 3B). The peak of ArE probability curves notably shifted rightwards in parallel with an increase in NYHA functional class. The ratio of ArE to HFD increased proportionally with MIBG HMR in each NYHA category (Figure 4). The increasing trend of ArE probability was more evident when NYHA functional class decreased.

**Figure 5:**
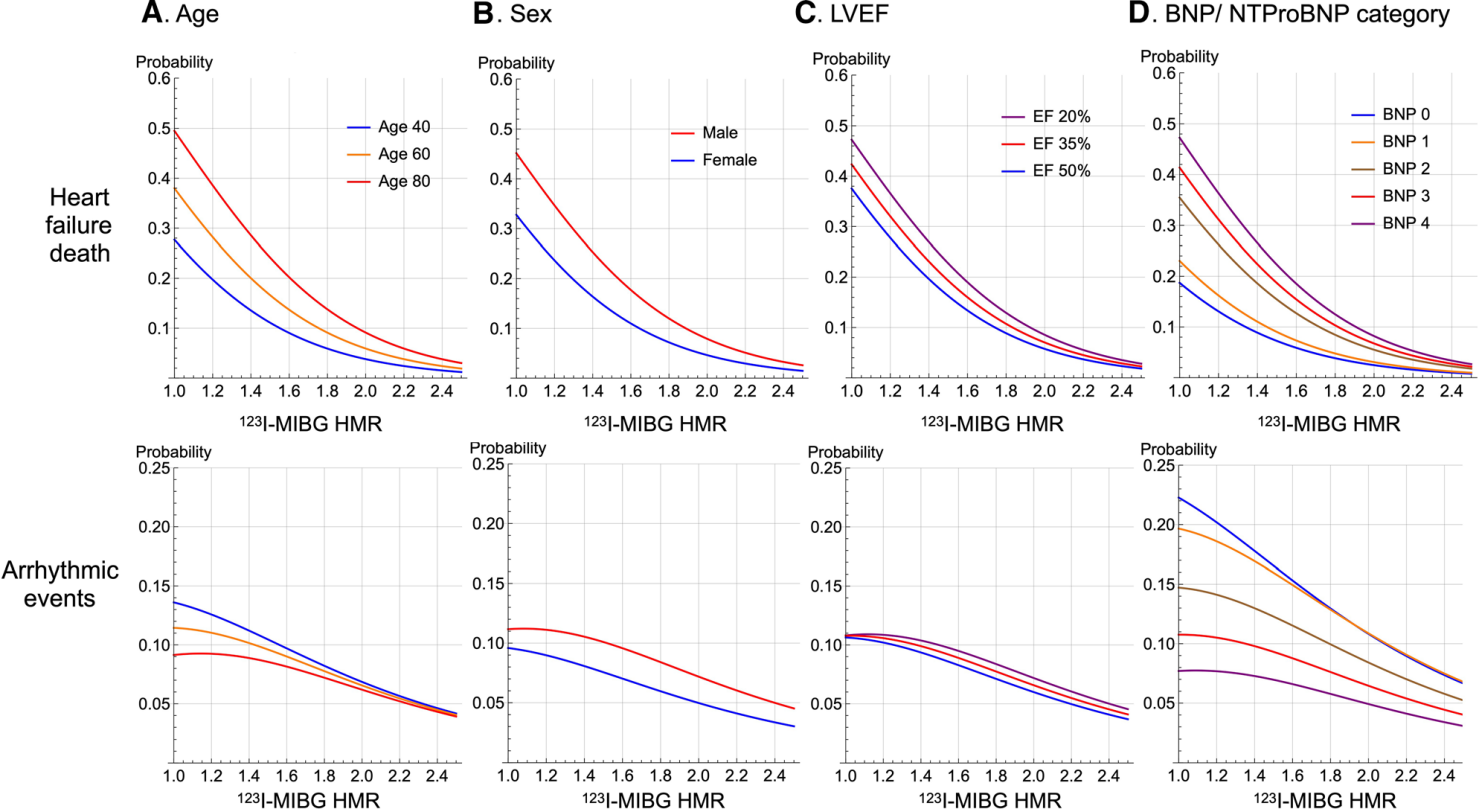
Probabilities of heart failure death and arrhythmic events plotted against ^123^I-MIBG heart-to-mediastinum ratio (HMR) in patients aged 40, 60 and 80 years (**A**), male and female (**B**) patients with different LVEF (**C**) and BNP category (**D**)

The probability of HFD increased inversely with ^123^I-MIBG HMR among elderly patients, whereas younger patients tended to have a greater probability of ArE at an MIBG HMR range < 1.6 (Figure 5A). Compared with females, male patients had a greater prevalence of cardiac mortality due to both HFD and ArE (Figure 5B). When LVEF was categorized as 20%, 35% and 50%, the effect of LVEF was small, and ^123^I-MIBG HMR was much more closely associated with the probability of cardiac death (Figure 5C). A higher BNP grade increased the probability of HFD but decreased that of ArE (Figure 5D).

**Figure 6:**
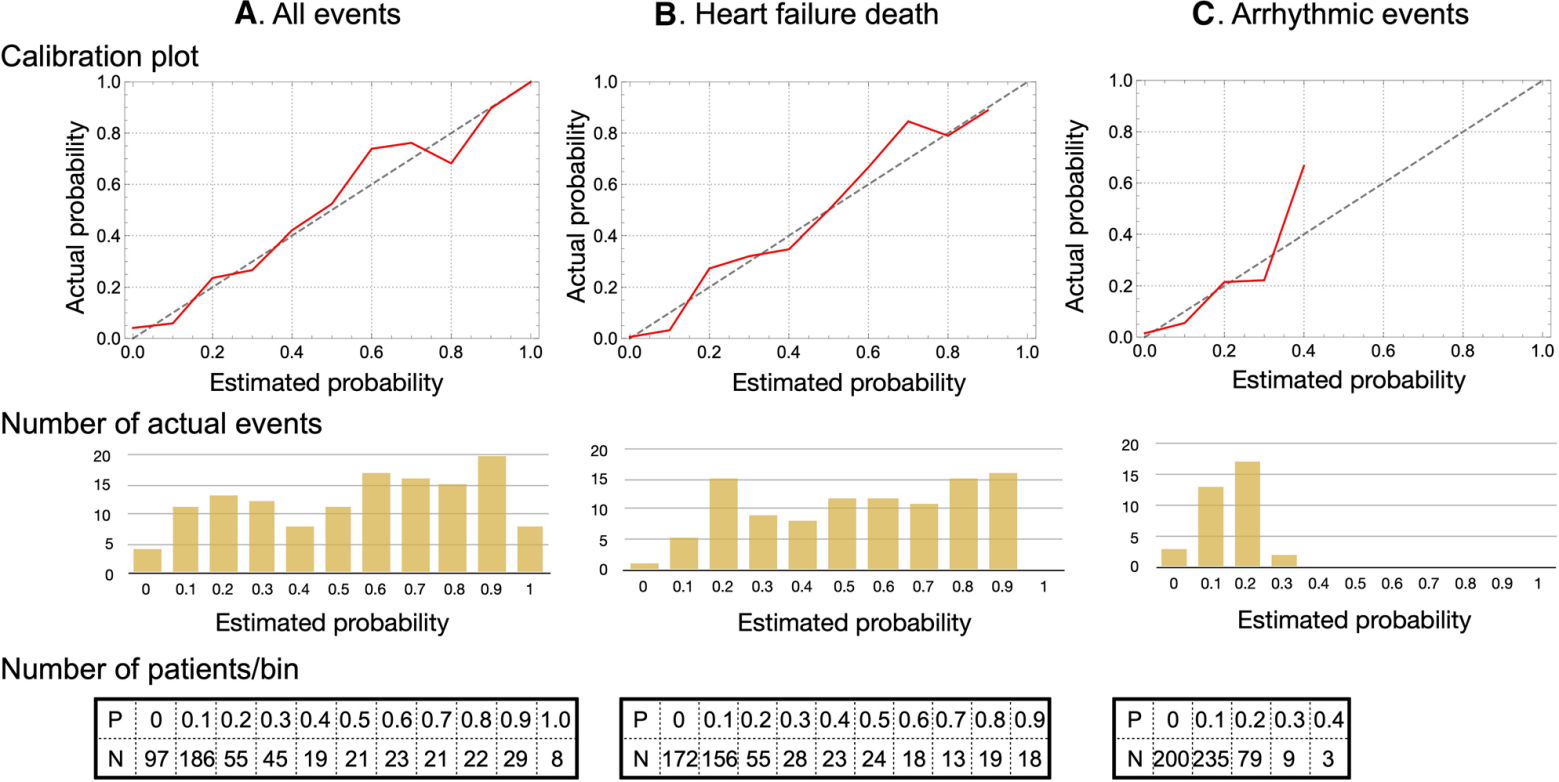
Calibration plots for all events (**A**), heart failure death (**B**) and arrhythmic events (**C**). Number of patients in each bin and actual number of events shown at bottom

The AUC of the logistic regression-based ROC curves used for these probability calculations were 0.88, 0.92 and 0.80 for all events, HFD and ArE, respectively. Calibration plots showed that the classifier was unbiased, or calibrated well, for estimated probability (Figure 6).

**Figure 7:**
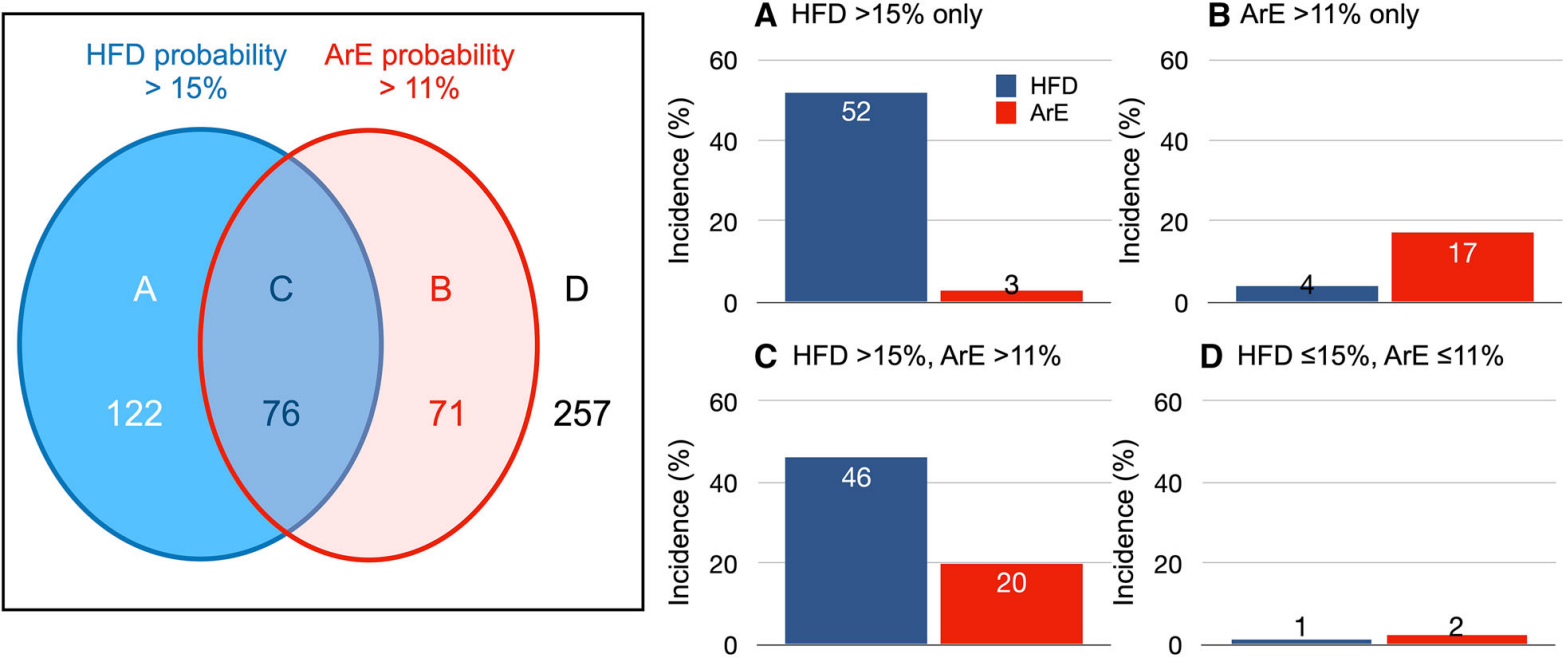
Patients with high and low probabilities of HFD and ArE, and actual incidence of HFD and ArE in each group. Estimated probability groups are as follows: HFD > 15% and ArE ≤ 11% (**A**), ArE > 11% and HFD ≤ 15% (**B**), both HFD > 15% and ArE > 11% (**C**), and HFD ≤ 15% and ArE ≤ 11% (**D**)

The optimal cutoff probability with the highest sum of sensitivity and specificity in each cardiac death mode was 15% for HFD and 11% for ArE. At an HFD cutoff of 15%, actual event rates were 2% (6/328) for the low-risk category with ≤ 15% probability and 49% (98/198) for the high-risk category with a probability ≥ 15% (*P* < .0001). At an ArE cutoff of 11%, the actual event rates were 3% (10/379) for the low-risk category with ≤ 11% probability and 18% (27/147) for the high-risk category with a probability > 11% (*P* < .0001). Figure 7 demonstrates combinations of high and low probabilities for HFD and ArE and actual incidence of HFD and ArE documented during the follow-up. The patients with high HFD > 15% and ArE > 11% showed respectively high HFD and ArE events, whereas patients with a low probability of HFD ≤ 15% and ArE ≤ 11% showed very low event rate (*P* < .0001).

## Discussion

We differentiated risk for HFD and serious arrhythmic events using a multicenter database of CHF, ^123^I-MIBG and a machine learning-based classifier. Although conventional statistical models can predict cardiac mortality risk using ^123^I-MIBG, risk for ArE and HFD could not be clearly separated.^8^,^9^,^19^ The machine learning-based approach was effective in the face of multifactorial prediction models.

### Machine Learning

The machine learning approach is becoming more prevalent within the medical community, particularly within the domain of cardiovascular diseases and cardiac imaging.^20^ This approach can also be applied together with conventional statistical methods to analyze non-imaging clinical databases. For example, the diagnostic ability of a machine learning algorithm combined with automated perfusion quantitation software and clinical variables was comparable to or better than that of experts in terms of nuclear cardiology diagnoses.^21^ When an artificial neural network analysis was used for myocardial perfusion imaging to identify ischemia and/or infarction, the diagnostic accuracy was more effective than conventional defect scoring using dedicated nuclear cardiology software.^22^ Moreover, machine learning could be applied to predict major adverse cardiac events when combined with both clinical and imaging data variables.^23^ Various machine learning methods can be used to obtain appropriate classifier models, and the optimal method for any purpose can be selected.

Univariate and multivariate analyses are usually applied in conventional statistical analysis, and variables with good probability (usually *P* < .05) can be selected. We selected age, gender, NYHA class, LVEF and ^123^I-MIBG HMR to create models that could predict 2- or 5-year cardiac death.^8^,^19^ However, ArE was impossible to predict, because no single variable was useful.

Another viewpoint is how the combined effects of variables on target events could be analyzed. Even when each variable is insignificant in terms of event prediction, combined morbidities such as diabetes, chronic kidney disease and hypertension might synergistically increase event risk. Such collective effects could be more easily simulated by machine learning-based models, because they are better suited to finding nonlinear relationship between inputs and outputs.

Since the present study aimed to create a classifier function to evaluate associations between ^123^I-MIBG HMR and event rates, we selected relatively simple logistic sigmoid regression, which provided stable probability curves. We also tested a neural network approach in a preliminary evaluation, but it was liable to result in overfitting during training and thus it was not used to prepare the classifier in the present study.

### Risk of Heart Failure Death and Arrhythmic Events

Characteristics curves of relationships differed when the probabilities of HFD and ArE were plotted against HMR. The ADMIRE-HF study using the multivariate Cox proportional hazards model identified LVEF, BNP and ^123^I-MIBG defect scores as predictors of time to an arrhythmic event and related an intermediate reduction in ^123^I-MIBG activity to a higher likelihood of arrhythmic events.^9^ Five-year follow-up by ADMIRE-HF showed that patients with preserved sympathetic innervation (^123^I-MIBG HMR > 1.60) were at significantly lower risk of cardiac death, arrhythmic events, sudden cardiac death, or potentially life-threatening arrhythmias, but whether these risks were evident at intermediate HMR was not documented.^24^,^25^ European multicenter studies of patients with prophylactic ICD implantation independently associated late HMR with combined endpoints such as appropriate ICD therapy, progression of heart failure, and cardiac death, and found that ICD therapy was appropriate in the intermediate HMR range.^26^,^27^

Our machine learning-based modeling showed that risk for ArE was the highest at the intermediate range of HMR in association with NYHA class. The peak of the bell-shaped curve for ArE probability vs HMR shifted rightwards with increasing NYHA class. Since ADMIRE-HF and European studies enrolled only patients with NYHA functional classes II and III, the characteristics of this bell-shaped correlation was only partly evident.^7^,^26^,^27^ In contrast, the probability of ArE consistently increased relative to a decrease in cardiac ^123^I-MIBG HMR in patients with NYHA functional classes I and II.

The increased risk of ArE at intermediate ^123^I-MIBG can be explained by the arrhythmogenicity of the injured/denervated but viable myocardium. An imbalance between preserved myocardial perfusion and impaired sympathetic innervation is the most likely pathophysiological cause of serious arrhythmias. A regional mismatch between myocardial perfusion and ^123^I-MIBG uptake is associated with ventricular arrhythmias, and large ^123^I-MIBG defects are significantly related to more appropriate ICD therapy.^28^ Such a mismatch might indicate that deranged metabolic activity and/or denervated hypersensitivity are responsible for serious arrhythmias in the injured, but viable myocardium. In contrast, advanced pathophysiology with absolute denervation and total necrosis or fibrosis might be less arrhythmogenic, thus blunting the correlation between ArE risk and cardiac ^123^I-MIBG activity. Patients with advanced heart failure are far more likely to experience progressive heart failure leading to pump failure death rather than arrhythmic events at a relatively earlier stage of the clinical course. Thus, in contrast to the consistent correlation between the likelihood of HFD and cardiac ^123^I-MIBG activity (HMR), ArE risk was less likely at a low HMR range of cardiac ^123^I-MIBG activity.

### Clinical Implications

Patients with CHF at increased risk for fatal cardiac events need an effective prophylactic strategy. The potential value of ^123^I-MIBG for predicting a need for ICD has also been evaluated.^28^-^33^ For this purpose, the accurate identification of responsible cardiac risks and of which is the most involved in the mode of cardiac death of individual patients with CHF is critical. The present study showed that ArE was more likely to develop in younger patients with less severe heart failure and moderately reduced ^123^I-MIBGactivity, whereas HFD was more frequent in older patients with a worse NYHA class, comorbidities and far less ^123^I-MIBG activity. Besides information about cardiac sympathetic nerve function, these features were similar to the clinical observations of the ESC-Failure Pilot study.^2^ In this study, sudden cardiac death was more prevalent among younger male patients with a better NYHA functional class. In contrast, pump failure death was more prevalent among older patients who had more symptoms, a worse NYHA class and/or non-cardiac comorbidities.

Although multiple factors are involved in the development of fatal outcomes, cardiac sympathetic innervation assessed by neuroimaging tracers has the potential to identify patients at increased risk of sudden/arrhythmic death who are likely to benefit the most from appropriate ICD treatment.^34^ Several ^123^I-MIBG studies have identified a significant incremental prognostic value of ^123^I-MIBG together with clinical information for the overall cardiac mortality of patients at low and high risk.^10^,^19^,^35^ Based on current indication criteria for ICD and CRT, a significant number of ICD devices are unlikely to deliver appropriate therapy during the lifetimes of patients, and about one-third of patients under CRT will succumb to cardiac death while under treatment with ineffective devices.^36^ Device-related issues and unrequited medical costs for such patients can reduce the cost-effectiveness of device therapy, indicating the need to establish more appropriate identification of those who are most likely to benefit from it in a cost-effective fashion.

Positron tracers such as ^11^C-hydroxyephedrine (HED)^37^ and ^18^F-labeled norepinephrine transporter (LMI1195)^38^ have superior image quality and quantitative accuracy to single-photon ^123^I tracers. In ischemic cardiomyopathy the potential utility of ^11^C-HED to identify patients most likely benefit from ICD therapy has been investigated (PARAPET study). Whether these new radiotracers have roles in risk stratification in conjunction with machine learning needs to be evaluated.

## Limitations

This study used a CHF database that were retrospectively created by combining medical charts from four hospitals. Cutoffs for risk stratification of ArE can be influenced by the database used for machine learning. Inclusion of acute myocardial infarction, which was not included in this database, may enhance the clinical applicability in CHF patients. However, although MIBG studies may be indicated in patients with CHF, it is not usually indicated to those who have high likelihood of acute myocardial infarction in clinical practice. A prospective cohort study using more accurate clinical information and outcomes is desirable to improve the predictive accuracy of the risk model. The low (7%) event rate of ArE might have been insufficient for clinically reliable analysis, indicating a need for a larger and more long-term cohort study to develop a high-performance risk model. Finally, a prospective interventional study is required to establish not only the clinical implications of machine learning-based risk assessment, but also risk-based therapeutic strategies.

## New Knowledge Gained

Based on machine learning, the likelihood of death from heart failure and fatal arrhythmic events can be arbitrarily simulated by the probability function, and relationship between ^123^I-MIBG HMR and fatal events can be estimated. The probability of fatal arrhythmic events was separately determined for the first time.

## Conclusion

We differentiated serious arrhythmias from end-stage heart failure as adverse cardiac event risks using a machine learning-based prognostic model created using variables that included cardiac ^123^I-MIBG activity, LVEF, NYHA class, age, gender, and other clinical variables. Our findings revealed differences in the probabilities of these two modes of cardiac death as well as in the pathophysiology of lethal cardiac events in chronic heart failure. Therefore, this information should contribute to more precise selection of prophylactic strategies tailored to the risk status of individual patients.

## Electronic supplementary material

Below is the link to the electronic supplementary material.

Electronic supplementary material 1 (PPTX 2593 kb)

## Abbreviations

ArE: Arrhythmic event
BNP: b-Type natriuretic peptide
CHF: Chronic heart failure
CRT-D: Cardiac resynchronization therapy with defibrillator
HFD: Heart failure death
HMR: Heart-to-mediastinum ratio
ICD: Implantable cardioverter defibrillator
LVEF: Left ventricular ejection fraction
MIBG: Metaiodobenzylguanidine
NT-ProBNP: N-terminal proBNP
NYHA: New York Heart Association
