# Supplementary figures and images for: Machine learning-based risk model using 123I-metaiodobenzylguanidine to differentially predict modes of cardiac death in heart failure
Source: J Nucl Cardiol. 2020 May 14;29(1):190–201. doi: 10.1007/s12350-020-02173-6 (PMC8873155; doi:10.1007/s12350-020-02173-6)

## Slide 1
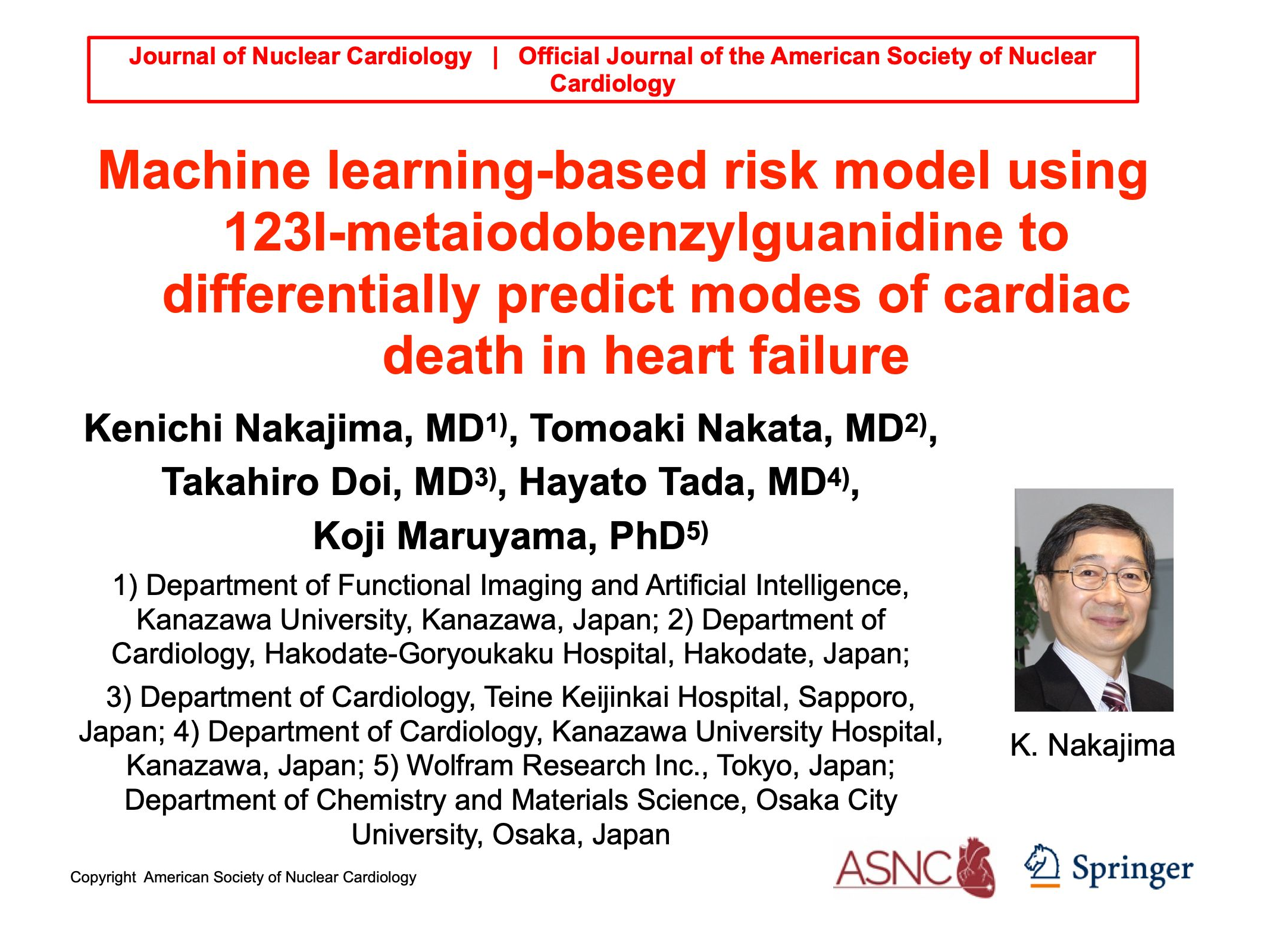

## Slide 2
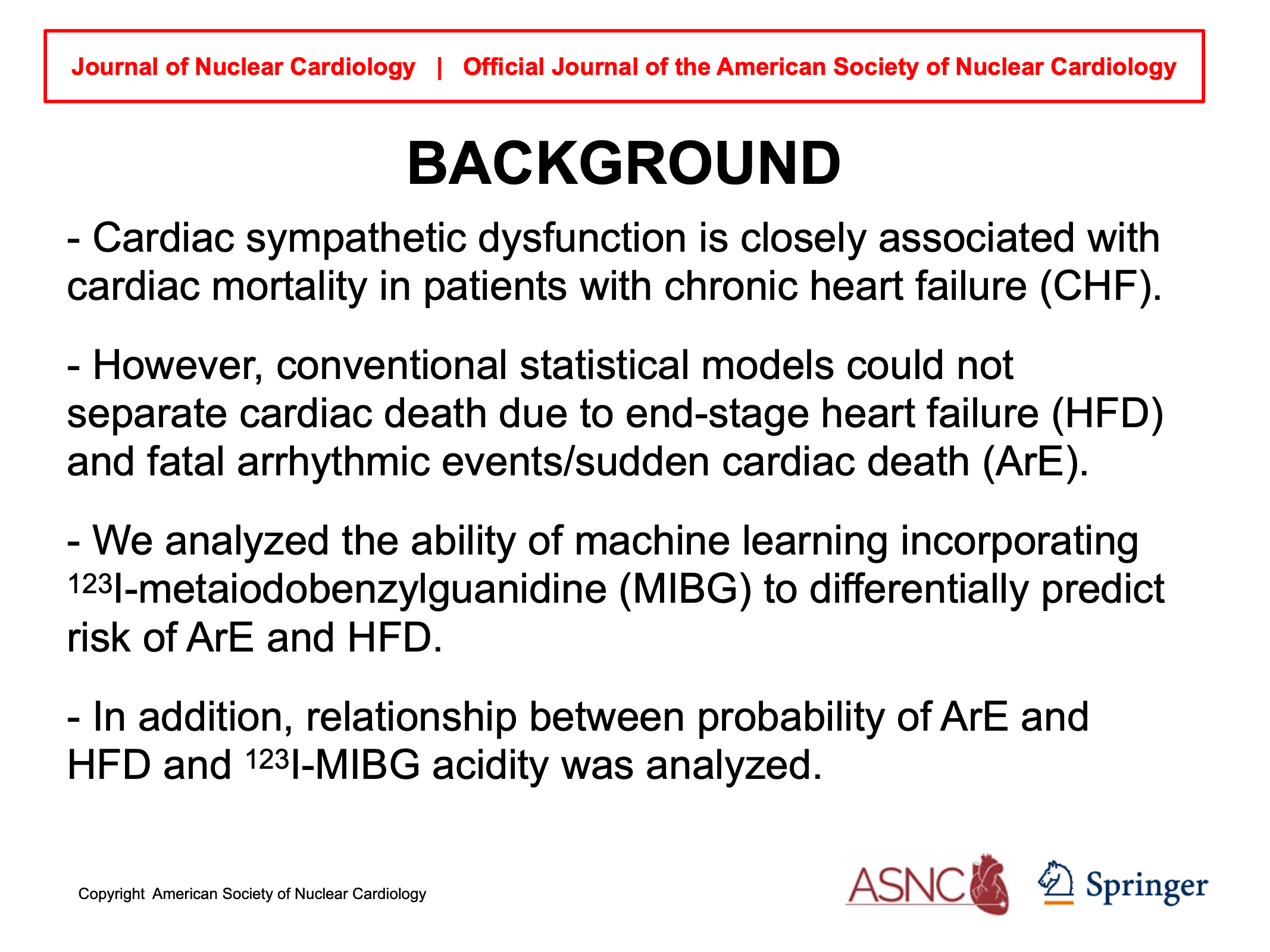

## Slide 3
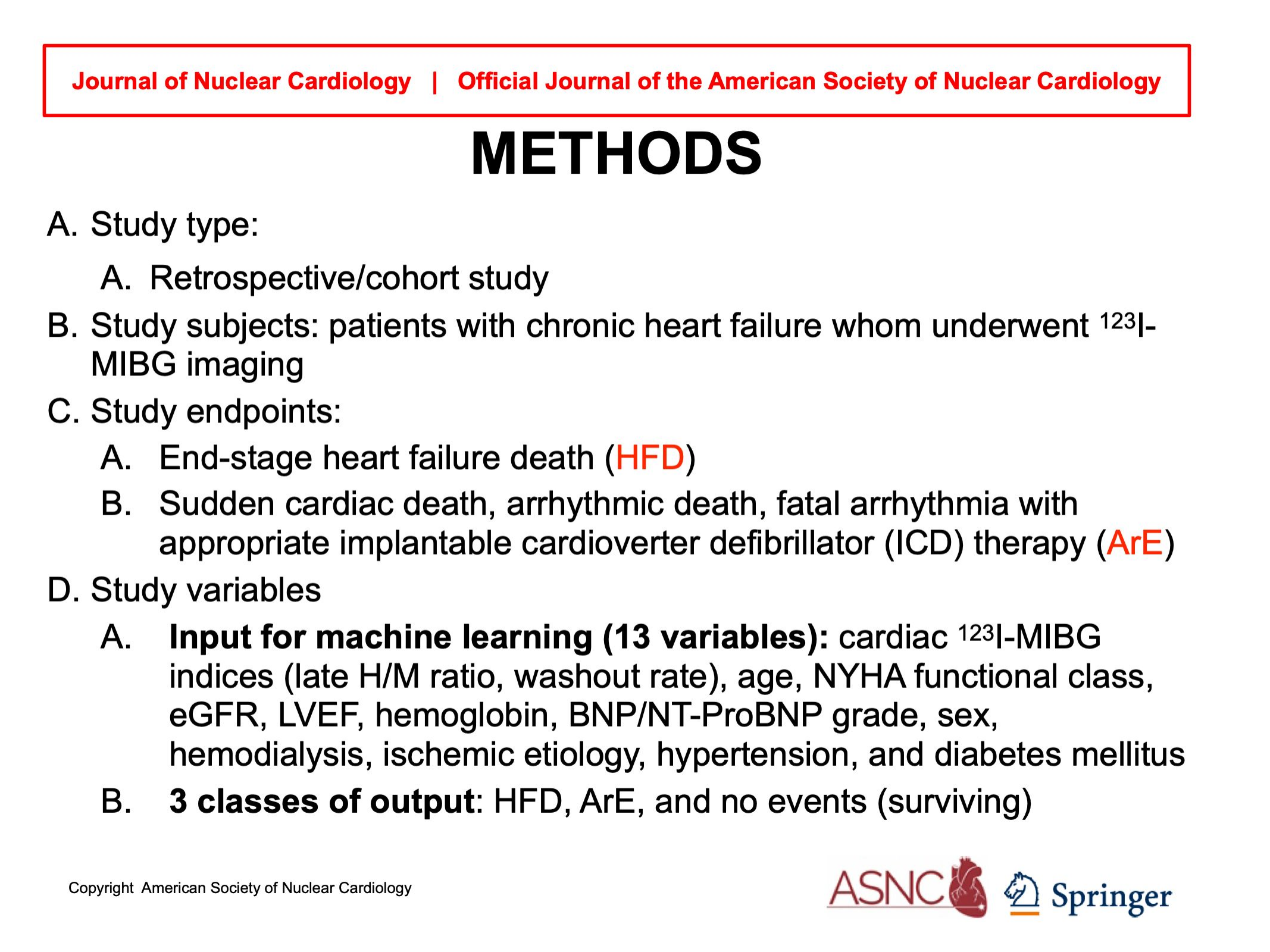

## Slide 4
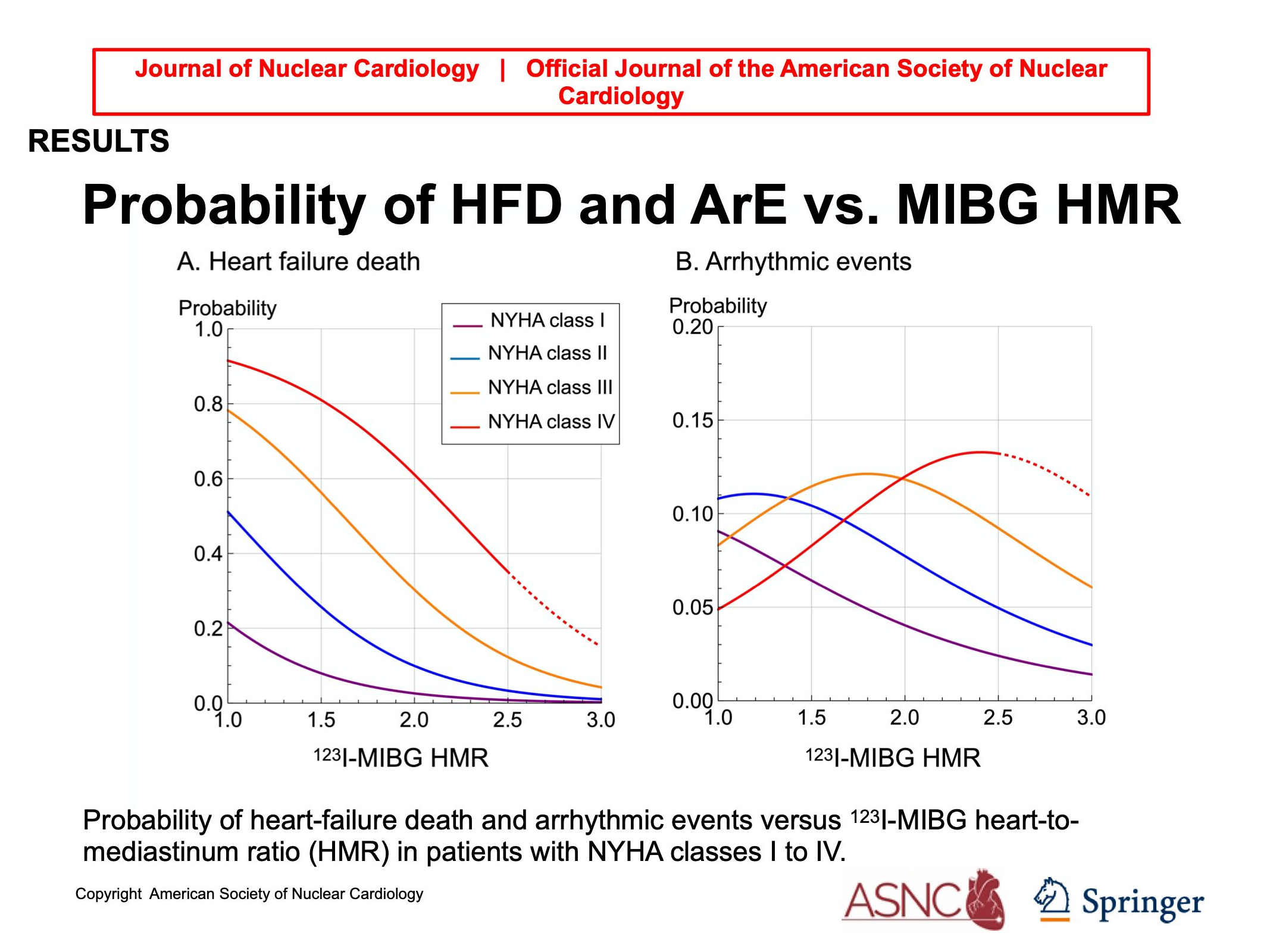

## Slide 5
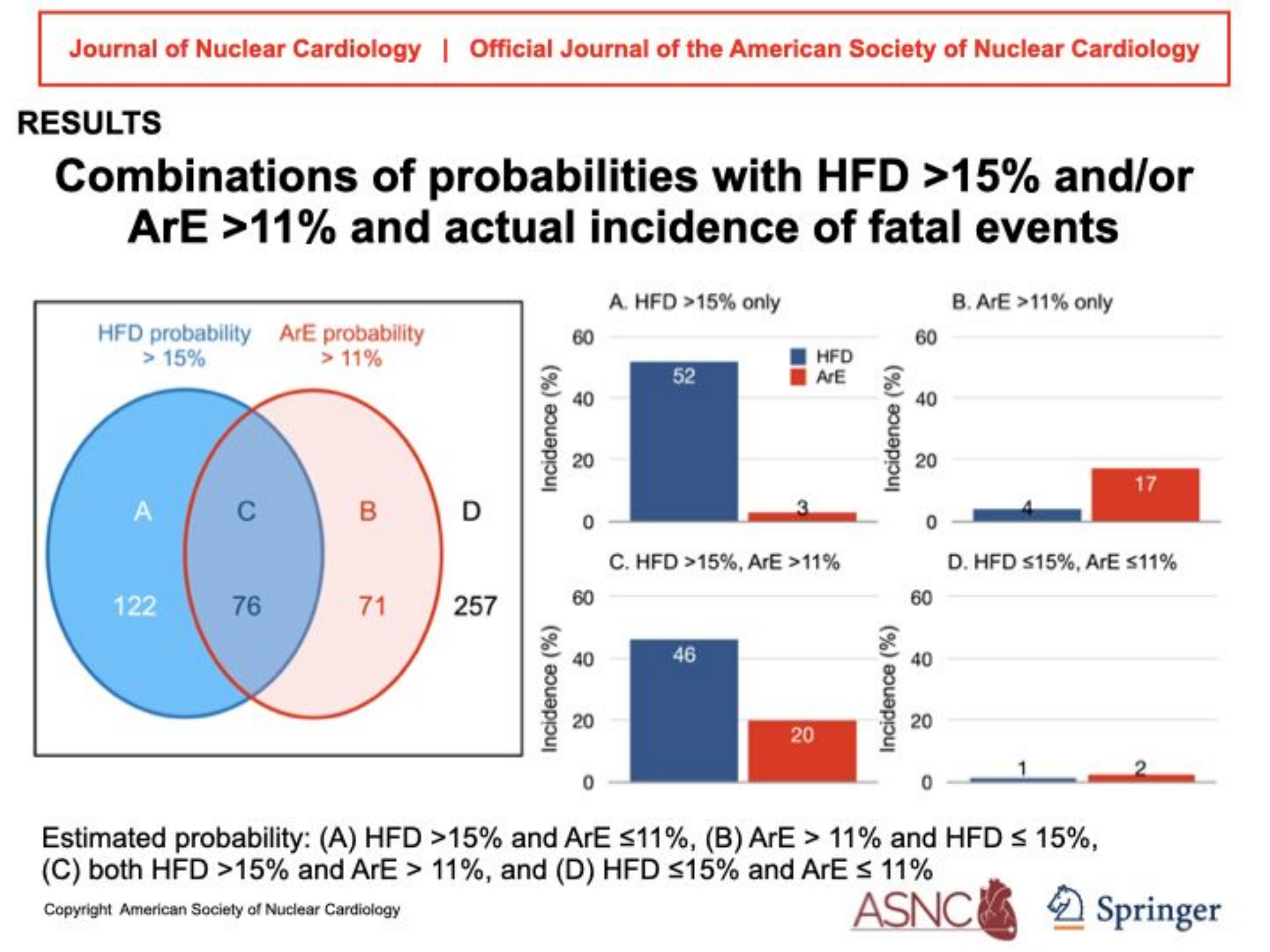

## Slide 6
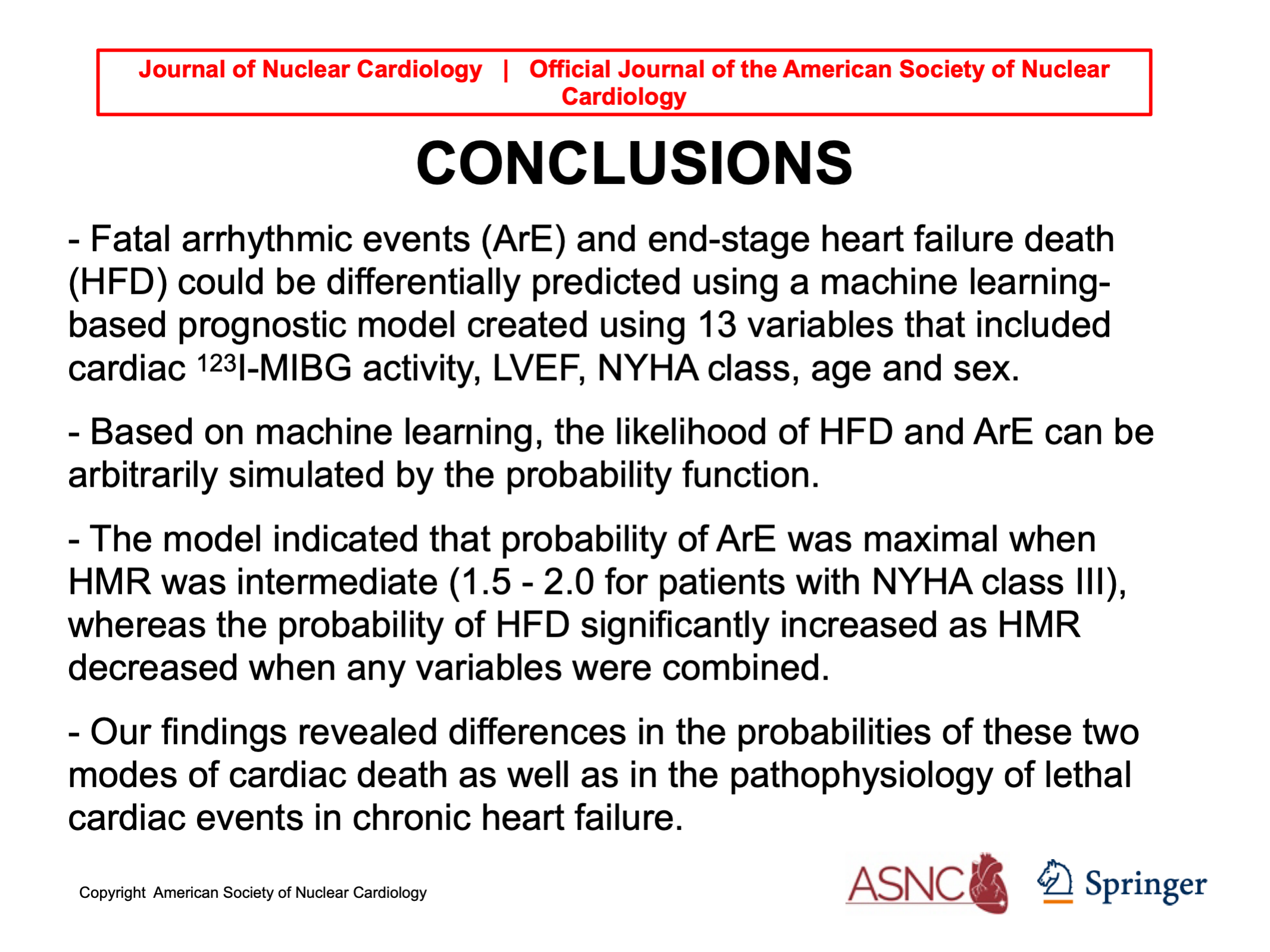

Supplement: Supplementary file 1 — Electronic supplementary material 1 (PPTX 2593 kb) [file 12350_2020_2173_MOESM1_ESM.pptx]
